# Mendelian randomization: genetic anchors for causal inference in epidemiological studies

**DOI:** 10.1093/hmg/ddu328

**Published:** 2014-07-04

**Authors:** George Davey Smith, Gibran Hemani

**Affiliations:** MRC Integrative Epidemiology Unit (IEU) at the University of Bristol, School of Social and Community Medicine, Bristol, UK

## Abstract

Observational epidemiological studies are prone to confounding, reverse causation and various biases and have generated findings that have proved to be unreliable indicators of the causal effects of modifiable exposures on disease outcomes. Mendelian randomization (MR) is a method that utilizes genetic variants that are robustly associated with such modifiable exposures to generate more reliable evidence regarding which interventions should produce health benefits. The approach is being widely applied, and various ways to strengthen inference given the known potential limitations of MR are now available. Developments of MR, including two-sample MR, bidirectional MR, network MR, two-step MR, factorial MR and multiphenotype MR, are outlined in this review. The integration of genetic information into population-based epidemiological studies presents translational opportunities, which capitalize on the investment in genomic discovery research.

## INTRODUCTION

Many examples exist of apparently robust observational associations between behavioural, pharmacological or physiological measures and disease risk which, when subjected to randomized controlled trials (RCTs), do not deliver the anticipated health benefits (1). These include many nutritional factors (e.g. several vitamins), pharmacological agents (e.g. hormone replacement therapy) and circulating biomarkers (e.g. HDL cholesterol) (1–4). Confounding, reverse causation and various biases can generate the associations, and even with careful study design and statistical adjustment, incorrect causal inference is possible (1,5). The recognition of these problematic aspects of epidemiological investigation has led to the application of a series of methods aimed at improving causal inference (6,7). A successful approach is to use genetic variants as exposure indicators that are not subject to the influences that vitiate conventional study designs, an approach known as Mendelian randomization (MR) (8,9). We will not repeat the many detailed reviews that now exist of MR (8,10–15) nor summarize the hundreds of empirical studies applying the technique to a wide range of exposures and disease outcomes, rather, after a brief summary of the foundational principles, we will outline recent developments and potential future directions of the field.

## BASIC PRINCIPLES OF MENDELIAN RANDOMIZATION

**Figure 1.**
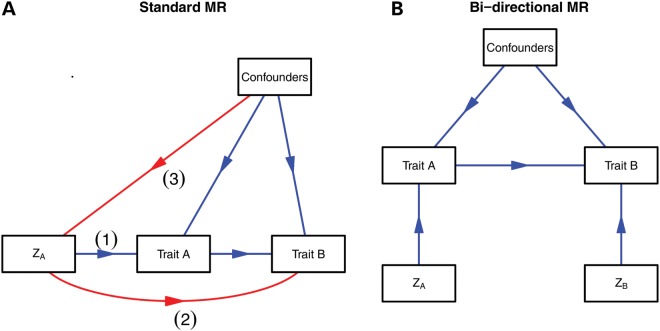
Schematic representation of MR. (**A**) Mendelian randomization can be used to test the hypothesis that trait A causes trait B, provided that conditions (1), (2) and (3) are met adequately, governing that *Z*_A_ is a valid instrument, in that (1) it is associated with the intermediate phenotype of interest; (2) has no association with the outcome except through the intermediate phenotype, and (3) is not related to measured or unmeasured confounding factors. (**B**). In bi-directional MR, the causal direction between traits (A and B) (if any) can be elucidated, if valid instruments are present for each trait.

Inferring the causal direction between correlated variables is a pervasive issue in biology that simple regression analysis cannot answer. The association between two variables could reflect a causal relationship, but the direction of causality (e.g. A causing B or B causing A) is not clear. Furthermore, there may be unobserved factors that influence both variables and lead to their association (confounding) (Fig. 1). In the latter scenario, the effect of the independent variable on the outcome may be zero. Even if the hypothesized causal direction were correctly specified, if the independent variable is correlated with some unobserved or imprecisely measured confounders then the estimate of its causal effect could be biased. Mendelian randomization is a technique aimed at unbiased detection of causal effects and, where possible, estimation of their magnitude.

Suppose that trait A and trait B are correlated, it follows that if this correlation arises because A is causing B, then any variable that influences trait A should also influence trait B. The key to inferring a causal relationship between A and B is to identify an ‘instrument’ that is reliably associated with A in a known direction. Biologists are in a privileged position in this regard because virtually all traits of interest are at least partially influenced by genetic effects, and genetic effects can serve as excellent instruments for a number of reasons. First, in a genetic association, the direction of causation is from the genetic polymorphism to the trait of interest, and not *vice versa*. Second, conventionally measured environmental exposures are often associated with a wide range of behavioural, social and physiological factors that confound associations with outcomes (16). Genetic variants, on the other hand, can serve as unconfounded indicators of particular trait values (16). Third, genetic variants and their effects are subject to relatively little measurement error or bias. Fourth, the actual causal variant for the trait is not required, a marker in linkage disequilibrium (LD) with the causal variant will satisfy the conditions for MR. Finally, in the era of genome-wide association studies (GWAS) and high-throughput genomic technologies, genetic data are routinely available on large well-phenotyped studies.

## ANALOGY BETWEEN MENDELIAN RANDOMIZATION AND RANDOMIZED CONTROLLED TRIALS

An intuitive way to understand how MR can be used to infer causality is by analogy with RCTs. In RCTs, the study participants are randomly allocated to one or another treatment, avoiding potential confounding between treatment and outcome, and causal inference is unambiguous. MR creates a similar scenario for us. Suppose a particular allele is robustly related to trait A, and trait A causes trait B. Alleles are largely passed from parents to offspring independent of environment, and people who inherit the allele are, in effect, being assigned a higher on-average dosage of trait A, whereas those who do not inherit the allele are assigned a lower on-average dosage. As in RCTs, groups defined by genotype will experience an on-average difference in exposure to trait A, whilst not differing with respect to confounding factors. Thus, a by-genotype analysis is equivalent to an intention-to-treat analysis in a RCT, in which individuals are analysed according to the group they were randomized into, independent of whether they complied to the treatment regimen or not. This form of analysis ensures that confounding is not reintroduced though allowing reclassification of exposure status after randomization.

Empirical evidence that there is a general lack of confounding of genetic variants with factors that confound exposures in conventional observational epidemiological studies is extensive (16,17), although it is important to take appropriate measures to avoid introducing confounding through population stratification.

To date, MR has been successfully applied to a wide range of observational associations, covering applications to the causal effects of biomarkers on disease, understanding the correlation between physiological measures, estimating the causal effects of various behaviours and specifying maternal intrauterine influences (Table 1). In certain circumstances, it is possible to perform an instrumental variable analysis to obtain an estimate of the magnitude of the causal effect of the exposure of interest on the outcome under investigation, and we outline this in Box 1. There are a number of limitations to MR that should be considered when using this approach (Table 2), which have been discussed at length elsewhere (8,10–15). Pleiotropy (Box 2) is particularly problematic in this regard. The remainder of this review will outline recent developments in MR, some of which explicitly seek to address these limitations.

**Table 1.** Examples of MR

Type	Exposure/trait	Disease/outcome	Conclusion
Biomarkers	CRP	Coronary heart disease	Observational association between CRP and coronary heart disease is a result of confounding and/or reverse causation (18)
	Serum iron	Parkinson's disease	Higher serum iron levels lower the risk of Parkinson's disease (19)
	Uric acid	Coronary heart disease	Observational association between uric acid and coronary heart disease is, in part, due to confounding by BMI (20)
	Macrophage migration inhibitory factor (MIF)	Type 2 diabetes	Elevated MIF, amongst other factors, increases the risk of type 2 diabetes (21)
	Interleukin 6 (IL6)	Coronary heart disease	IL6 increases the risk of coronary heart disease (22,23)
Behaviours	Smoking	Anxiety/depression	Anxiety and depression amongst smokers does not appear to be a consequence of smoking (24,25)
	Alcohol consumption	Blood pressure	Alcohol use increases blood pressure (26)
Physiological measures	BMI	Symptomatic gallstone disease	Higher BMI increases the risk of symptomatic gall stone disease (27).
Maternal influences (corrected for genetic correlation between mother and child)	Alcohol consumption	Childhood school performance	The observational finding that moderate maternal alcohol intake is associated with more favourable school performance is due to confounding, and the casual association is in the opposite direction (28)
	Maternal BMI	Fat mass of offspring	Fat mass in children aged 9–11 is not strongly influenced by BMI of mothers during pregnancy (29)

**Table 2.** Limitations of MR

Limitation	Role in MR studies	Approaches to evaluating or avoiding the limitation
Low statistical power	MR studies are often of low power and effect estimates are imprecise because of this	Increase sample size and or combine genetic variants so they explain more of the variance of the intermediate phenotype
Reverse causation	A genetic variant may be causing the disease outcome which in turn causes the biomarker, or the causal direction could be in the opposite direction. 2SLS will not distinguish between these cases	Bi-directional MR can be used to distinguish between the two causal models
Population stratification	Spurious associations used as instruments can lead to faulty causal inference	Restrict analyses to ethnically homogeneous groups, and apply correction methods using ancestrally informative markers or principal components from genome-wide data. Perform analysis within a family study context, e.g. between siblings.
Reintroduced confounding though pleiotropy	A genetic variant may directly influence more than one post-transcriptional process. Known to be the case for some genetic variants	When possible utilize *cis*-variants with respect to the intermediate phenotype under study, as these may be less likely to have pleiotropic effects. Apply multiple instrument approaches with more than one independent genetic variant it is unlikely that pleiotropy will generate the same associations for different instruments
LD induced confounding	LD is crucial in genetic association studies as it allows marker SNPs to proxy for un-genotyped causal SNPs. However, this can reintroduce confounding if LD leads to the association of SNPs related to more than one post-transcriptional process. This case will be similar to the pleiotropy situation	Studies can be carried out in populations with different LD structures. Approaches to avoiding distortion by pleiotropy will also counter problems owing to LD
Canalization/developmental compensation	During development, compensatory processes may be generated that counter the phenotypic perturbation consequent on the genetic variant utilized as an instrument	No general approach developed, although context-specific biological knowledge can be applied. The period of the life course when influence of genetic variation on intermediate phenotypes emerge can indicate whether canalization could, in principle, be an issue
Lack of genetic variants to proxy for modifiable exposure of interest	No reliable genetic variant associations for many intermediate phenotypes of interest, although an increasing number of these now identified	Continued genome-wide and sequencing-based studies
Complexity of associations	Without adequate biological knowledge, misleading inferences regarding intermediate phenotypes and disease may be drawn	Increased biological understanding of genotype–phenotype links

Box 1. **Application of instrumental variable approaches to MR studies**

Conventional instrumental variable (IV) analysis requires that the instruments are valid, and in order to be valid, they must meet three conditions. An instrument for trait A must be:

1. reliably associated with trait A;

2. associated with the outcome (trait B) only through trait A and

3. independent of unobserved confounders that influence traits A and B after conditioning on observed confounders.

In MR, condition (1) is straightforward to test, but (2) and (3) cannot be established unequivocally. For example, if the variant is pleiotropic (see Box 2), or if it is in LD with a genetic variant that influences the outcome through a different mechanism, this can lead to erroneous causal estimation. If the above-mentioned conditions are met, then the unbiased estimate of the effect of trait A on the outcome, B, can be made using two-stage least-squares (2SLS) regression.

In stage 1, a predictor for A is constructed from its instrument, and in stage 2, the effect of the predictor for A on the outcome B is estimated. The intuition here is that A is potentially associated with B owing to many confounding effects, and we wish to estimate the effect of A on B that occurs only via the component of A associated with the instrument. Thus, if the predictor for A is associated with B in the estimate from stage 2, then this is only occurring through a path which has no confounding.

Several software implementations exist for performing various type of MR analysis. The ‘ivregress’ package in STATA, and the ‘systemfit’ package in R each have functions for performing 2SLS. The general case of IV estimation, including when the number of instruments is greater than the number of explanatory variables, can be performed using the generalized method of moments using the ‘gmm’ package in R (30). Few software examples exist for the specific types of MR that have been described in this review, but STATA routines for performing subsample and two-sample IV estimation are provided by Pierce and Burgess (31).

Box 2. **Consequences of pleiotropy for the interpretation of MR**

Pleiotropy is the phenomenon by which a single locus influences multiple phenotypes (32). Depending on the form it takes, pleiotropy may be a potential limitation to interpretation of MR, so distinguishing between its different types is important. In the context of MR, there are two mechanisms by which pleiotropy occurs: a single process leading to a cascade of events (e.g. a locus influences one particular protein product, and this causes perturbations in many other phenotypes); or a single locus directly influencing multiple phenotypes (33,34). Amongst its many names, the former has been termed ‘spurious pleiotropy’ (35,36), ‘mediated pleiotropy’ (37) or ‘type II pleiotropy’ (36); the latter ‘biological pleiotropy’ (37) or ‘type I pleiotropy’ (36). Type II pleiotropy is not only unproblematic for MR, it is the very essence of the approach, in which the downstream effects of a perturbed phenotype are estimated through the use of genetic variants that relate to this phenotype. Thus, the instrument of common variation in *FTO*, known to influence BMI (38), probably through influencing caloric intake (39,40), is associated with a wide range of downstream phenotypes; blood pressure and hypertension (41), coronary heart disease (42), fasting insulin, glucose, HDL cholesterol and trigylcerides (43), bone mineral density (44), chronic renal disease (45) and diabetes (38). These associations are expected, as higher BMI influences these traits, and it would be an error to consider these to be ‘pleiotropic’ effects of *FTO* variation that vitiate MR investigations.

Type I pleiotropy, however, is problematic for the interpretation of MR. Estimates of the degree of pleiotropy suggest that type II pleiotropy is the more pervasive form (36,46), with type I pleiotropy being more pronounced at the level of the gene than at the level of single SNPs (36,47). Greater pleiotropic effects are seen for mutations with larger effects on the primary trait (48,49), as would be anticipated for type II pleiotropic influences that are downstream effects of considerable perturbation of the primary trait.

Potentially erroneous causal inference owing to type I pleiotropy can be minimized by restricting instruments to genetic effects which plausibly act directly on the trait (e.g. genetic instruments for CRP levels located within the promoter region of the *CRP* gene). When less well-characterized variants, or combinations of variants, are utilized, then the ways of exploring the potential contribution of pleiotropy detailed in this review and elsewhere (15) need to be implemented.

## RECENT EXTENSIONS TO BASIC MENDELIAN RANDOMIZATION

### Use of multiple variants to increase power and test assumptions

Ideally, MR is performed using a single variant whose biological effect on the trait for which it is an instrument is understood. However, even this situation is subject to a few potential limitations, which can be partially mitigated by increasing the number variants used as instruments.

First, the genetic effect may not be particularly large, resulting in a weak instrument and the requirement for very large sample sizes. By increasing the number of variants, the proportion of variance explained by the instrument increases, thus improving precision in two-stage least-squares regression (Box 1) (50). Combining these into a weighted allele score is generally the optimal approach in this context (51).

**Figure 2.**
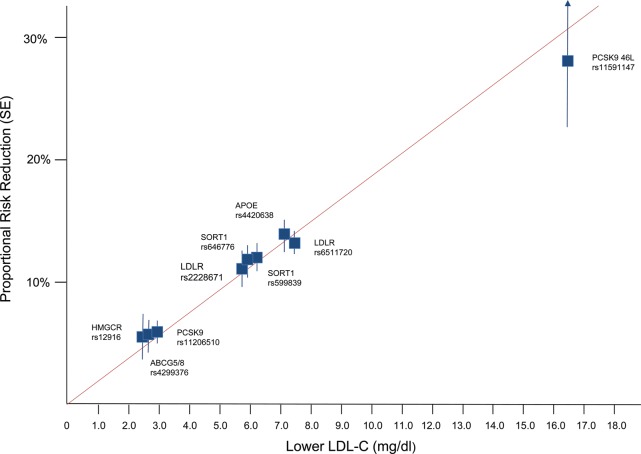
Effect of lower LDL-C on risk of CHD [taken from Ference *et al.* (2012) (52)]. Boxes represent the proportional risk reduction (1-OR) of CHD for each exposure allele plotted against the absolute magnitude of lower LDL-C associated with that allele (measured in mg/dl). SNPs are plotted in order of increasing absolute magnitude of associations with lower LDL-C. The line (forced to pass through the origin) represents the increase in proportional risk reduction of CHD per unit lower long-term exposure to LDL-C.

Second, the variant could be pleiotropic or in LD with a variant that affects the outcome, violating the conditions for being a valid instrument. This potential caveat can be interrogated by using multiple instruments. For example, it would be increasingly improbable that two, three or more independent instruments all result in the same conclusion, owing to perfectly balancing pleiotropic effects on both traits. For a convincing example demonstrating the causal influence of low-density lipoprotein cholesterol (LDL-C) on coronary heart disease (CHD), see Figure 2, where nine polymorphisms from six genes independently lead to very similar predicted causal effects of LDL-C, using instrumental variables analyses (52).

Third, multiple variants can also provide some evidence regarding the problematic issue of the complexity of associations in MR studies (see Box 3). If multiple variants that relate to a particular intermediate phenotype through different mechanisms all relate to the disease outcome in the manner predicted by their association with the intermediate phenotype—as in the case of multiple variants related to LDL-C and CHD, discussed earlier—the particular way through which one variant relates to the intermediate phenotype is unlikely to influence the cumulative evidence.

Box 3. **Complexity of associations**

In MR studies, genetic variants are taken to be proxy indicators of modifiable factors that potentially influence disease risk. The manner in which the variants relate to such factors can lead to misleading interpretations, however. For example, antioxidants are potentially protective against risk of CHD risk, so increasing circulating levels of the natural antioxidant extracellular superoxide dismutase (EC-SOD, a scavenger of superoxide anions), might be hypothesized to decrease CHD risk. However, a genetic variant associated with higher circulating EC-SOD is associated with substantially increased CHD risk (53). An explanation for this apparent paradox is that the genetic variant may influence circulating levels of EC-SOD by reducing the levels of EC-SOD in arterial walls; thus, the *in situ* anti-oxidative activity is lower, whereas the circulating levels are higher. A naive interpretation of the genetic studies—that higher levels of antioxidant increase risk of CHD—would be misleading. Similarly, it has been suggested that the interpretation of MR studies purporting to show that elevated uric acid levels do not increase risk of hypertension (20,54) is rendered problematic by the fact that the main genetic variant utilized in such studies, whilst increasing circulating uric acid levels, does not increase the intracellular level of uric acid, and the latter may be the important factor with respect to hypertension (55).

Typically, genetic variants are only used as instruments if they are reliably detected and replicated in GWAS. However, predictive power may be improved when SNPs that do not reach significance thresholds are also included, the rationale being that these will include false-negatives owing to small effect size (56). This approach can improve the power of MR, but considerable caution should be applied, owing to the increased chance of introducing pleiotropic effects (Box 2) (57).

### Two-sample Mendelian randomization

It is often the case that an observational association between two variables exists, but high measurement costs or lack of appropriate biospecimens leads to relatively small datasets with intermediate phenotypes and genetic instruments. Methods have been developed to perform IV analysis when the intermediate phenotype and the outcome variable are measured in two independent datasets (58), and these can be applied in the MR context (31). This approach can be particularly valuable when applied to the very large datasets that exist relating GWAS data to disease outcomes, but which lack intermediate phenotype data.

Another scenario in which two-sample MR can be used is if the dataset in which MR is being performed is the same as is being used to identify instruments. GWAS is known to lead to overestimation of genetic effect sizes owing to the phenomenon of the winner's curse, and this can lead to bias in MR. Dividing the dataset into two (or more) samples for estimation and testing can mitigate this problem. This method has been applied in a study of physical activity and childhood adiposity (59).

### Bidirectional and network Mendelian randomization

A major limitation of MR is that it can be difficult to distinguish between an exposure causing an outcome and an outcome causing a trait, because genetic variants could have their primary influence on either variable. For example, atheroma and body mass index (BMI) influence C-reactive protein (CRP) levels and apparent misleading causal effects can be generated if a genetic variant that primarily influences atheroma or BMI is mistaken as being a variant with a primary influence on CRP (60).

With a focus on instruments for which there exists some degree of biological understanding, bi-directional MR can be applied in these circumstances. Here, instruments are required for both variables, and MR is performed in both directions (Fig. 1). If trait A causes trait B, then the instrument, *Z*_A_, will be associated with both A and B. However, a second instrument specific to trait B, *Z*_B_, will be associated with trait B, and not with trait A. This method is only valid on the condition that the two instruments are not marginally associated with each other (e.g. there is no LD between instruments for A and B). This method has been used to demonstrate that BMI influences CRP levels (61,62), vitamin D (63), uric acid (20,64) and fetuin-A (65), and not *vice versa*. Extracting data from different studies can also be utilized in this context; for example, MR studies suggest that IL-6 influences CRP levels, but not *vice versa* (18,22,23).

When utilizing variants with little understanding of their biological effects, bidirectional MR can be potentially misleading, as it is obvious that if trait A influences trait B then GWAS studies with adequate statistical power will identify a variant with a primary influence on trait A as being associated with trait B. This reflects ‘spurious’ or ‘type II’ pleiotropy (Box 2), and many examples of this exist. For example, *FTO* variation was initially identified in relation to type 2 diabetes, with subsequent recognition that this was because the genetic variant related to BMI, which in turn increased the risk of type 2 diabetes (38). Similarly, genetic variants with a primary influence on BMI appear amongst the top hits in GWAS of CRP (66) but obviously cannot be utilized as instruments for CRP levels. Use of allele scores in bidirectional MR studies will increase the likelihood of incorrectly including a variant primarily influencing trait A as one that primarily influences trait B, with consequent misinterpretation, and findings from such studies need to be treated with caution (59). Utilizing multiple single and composite instruments can help interrogate such situations, because if trait A influences trait B, and not *vice versa*, then all variants related to trait A will relate to trait B, but the reverse will not be the case.

Bidirectional MR is applied in two-variable settings, but clearly this can be scaled up to explore the causal directions within a network of a larger number of correlated variables (67). Such ‘network MR’ is an area of current active development, with parallel logic to the application of genetic anchors in the causal dissection of networks of gene interactions (68,69).

### Mediation and two-step Mendelian randomization

Networks will often contain cases of mediation, in which the association between an exposure and an outcome may act through an intermediary factor. For example, higher BMI may increase the risk of CHD in part through its effect on blood pressure. Conventional mediation analysis in the epidemiological field, solely utilizing phenotypic measurements, is problematic, because it is highly dependent on the measurement characteristics of the variables and on reliable identification of causal effects (70–72). In such situations, it may be possible to obtain causal estimates from MR studies for all steps in the chain. In the above-mentioned example, MR studies have shown that greater adiposity leads to higher blood pressure (41), and in turn higher blood pressure increases the risk of coronary heart disease (73). More reliable specification of the quantitative contribution of the mediator (blood pressure) to the casual link between the exposure (BMI) and the outcome (CHD) could be made with such data.

MR approaches can be applied to mediation in situations of high-dimensional potential mediator data, as, for example, in the delineation of mediation by specific epigenetic processes between environmental exposures and disease. This has been referred to as two-step MR (74). Intermediate phenotypes, such as DNA methylation, can show tissue specificity, in that both genetic and phenotypic associations can differ between tissues, and assays of easily accessible samples (such as methylation of DNA extracted from blood) may not be representative of DNA methylation in the tissue that is responsible for disease development (75,76). Obtaining tissue-specific data on large numbers of individuals is challenging, but using a combined two-sample and two-step MR approach could be applied. First, the causal associations of both exposure on methylation and of a *cis* SNP on methylation in the tissue of interest could be established, and then in a larger population-based sample, the SNP associations with exposure and disease outcome delineated. Box 4 illustrates the logic of these more complex approaches.

Box 4. **Two-step and two-sample, two-step MR**

Genetic variants can be used as instrumental variables in a two-step framework to establish whether particular DNA methylation profiles are on the causal pathway between exposure and disease. In step 1, a SNP is used to proxy for the environmentally modifiable exposure of interest (e.g. smoking) to examine how this exposure influences DNA methylation. In step 2, a different SNP (which is not related to the exposure), preferably a *cis* variant, is used to proxy for this specific DNA methylation difference and to relate this to the disease outcome under investigation.

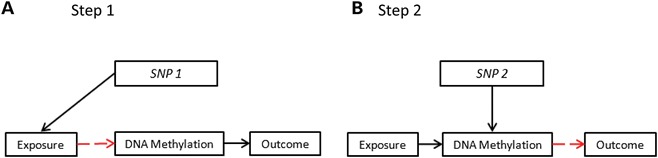

Two-sample, two-step MR can be utilized to interrogate tissue-specific DNA methylation as a potential causal intermediate phenotype. In the smaller first sample, the association of the exposure to tissue-specific DNA methylation is established using an MR approach (with the exposure-related SNP1; A) and a *cis* variant associated with the same methylation difference but not related to the exposure is identified (SNP2; B). In the larger second sample, the exposure is shown to influence the outcome through the use of SNP1, either through relating SNP1 to both the exposure (if data are available on this) and the outcome, or if exposure data are not available, then simply relating SNP1 to the outcome (C). Finally, exposure-related methylation is shown to influence the outcome through the use of SNP2, which is related directly to the outcome (D).

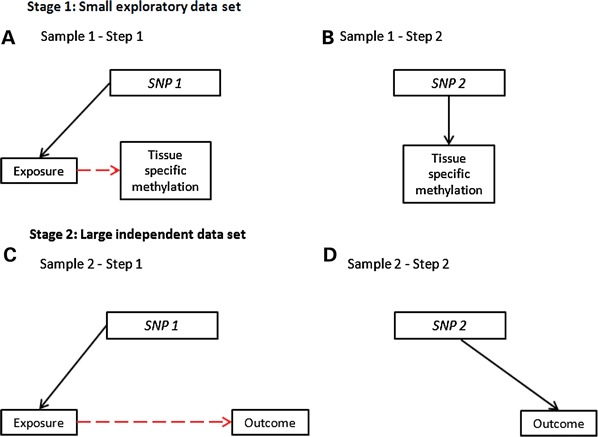

### Factorial Mendelian randomization

The manner by which causes of disease act together to increase disease risk can have important public health implications, as above-additive effects lead to the clustering of risk factors, generating a greater burden of disease in the population. For example, evidence exists that the combined influence of obesity and heavy alcohol consumption on the risk of liver disease is greater than multiplicative (77). It is difficult to estimate such effects, however, as confounding can be magnified when examining two already confounded risk factors. Factorial RCTs overcome this issue by randomizing each treatment independently, allowing characterization of interactions between them (78). Likewise, combinations of genetic variants can be used to perform factorial MR studies to obtain unconfounded estimates of the effect of co-occurrence of the two risk factors for disease.

### Multiphenotype Mendelian randomization

In some situations, genetic variants tend to be associated with multiple intermediate phenotypes, and estimating the causal effect of one particular intermediate phenotype is problematic. For example, HDL cholesterol and triglycerides are observationally associated with coronary heart disease, but they are also highly (inversely) correlated, and observational studies cannot reliably separate their effects (79). Many of the genetic variants related to HDL-C and triglycerides, of which there are a large number, associate with both measures (80), in what appear to be examples of type I pleiotropy (Box 2). Whereas factorial MR can be applied to multiphenotype relationships when different SNPs can be taken to be instrumental variables for each phenotype, in this case, this is not possible because constructing an instrument that purely relates to one phenotype is currently not possible. An initial way of interrogating this problem is to use regression methods to attempt to separate the effects, and two independent studies utilizing this approach have recently suggested that the causal influence of triglycerides was robust, whereas the apparent protective effect of HDL-C was not (81,82). The appropriateness of different statistical approaches and whether reliable answers can be obtained in the multiphenotype context remain areas of active investigation.

### Hypothesis-free Mendelian randomization

The majority of MR studies have been focused on testing hypotheses that arose from associations between traits seen in observational studies. But is this only the tip of the iceberg? An illustrative example of there being vastly more potential associations than those already known was presented by Blair *et al.* who, after mining the medical records of 110 million patients, uncovered 2909 associations between Mendelian diseases and complex traits, the majority of which were previously unreported (83). As high-throughput ‘omics technologies continue to reduce in time- and financial-cost, datasets with comprehensive genotyping and phenotyping are destined to grow, and in principle, it should be possible to construct instruments for many exposures and through data mining obtain evidence regarding outcomes caused by these exposures (57). More speculatively, generating instruments from within the data and performing split-sample or jackknife IV analysis, including bi-directional analysis, could allow resolution of causal direction within networks of phenotypes, without advance specification of which exposure or outcome is being examined (67).

### Conclusion

Resolving observational correlations into causal relationships is an elusive problem at the heart of biological understanding, pharmaceutical development, prevention of disease and medical practice. MR is a potentially robust method that can support this endeavour, and its scope for application will widen as the cost of data generation continues to reduce. Findings from MR studies need to be interpreted in the context of other evidence related to the particular issue under investigation, and as such, it will contribute to the application of ‘inference to the best explanation’ (84) approaches to strengthening causal inference. Identifying the most promising targets for intervention—for example, through pharmacotherapy—can be enhanced through the application of MR and thus lead to a more rational approach to prioritizing treatments for evaluation in RCTs.

## FUNDING

This work was supported by the Medical Research Council MC_UU_12013/1-9. Funding to pay the Open Access publication charges for this article was provided by the MRC Integrative Epidemiology Unit at the University of Bristol (MC_UU_12013/5).
